# The Association of Nephroblastoma Overexpressed (NOV) and Endothelial Progenitor Cells with Oxidative Stress in Obstructive Sleep Apnea

**DOI:** 10.1155/2021/7138800

**Published:** 2021-11-24

**Authors:** Eddie W. Fakhouri, Jeremy A. Weingarten, Shailendra P. Singh, Purvi Shah, Stephen J. Peterson

**Affiliations:** ^1^New York-Presbyterian Brooklyn Methodist Hospital, Brooklyn, NY 11215, USA; ^2^Department of Medicine, Weill Cornell Medicine, New York, NY 10065, USA; ^3^Department of Pharmacology, New York Medical College, Valhalla, New York 10595, USA

## Abstract

**Objective:**

Obstructive sleep apnea (OSA) is a sleep disorder characterized by intermittent hypoxia, chronic inflammation, and oxidative stress and is associated with cardiometabolic disease. Several biological substrates have been associated with OSA such as nephroblastoma overexpressed (NOV), endothelial progenitor cells (EPC), and circulating endothelial cells (CEC). Few studies have looked at the association of NOV with OSA while the EPC/CEC relationships with OSA are unclear. In this study, we hypothesize that (1) NOV is associated with the severity of OSA independent of BMI, identifying a protein that may play a role in the biogenesis of OSA complications, and (2) EPCs and CECs are also associated with the severity of OSA and are biomarkers of endothelial dysfunction in OSA.

**Methods:**

61 subjects underwent overnight polysomnography (PSG), clinical evaluation, and blood analysis for NOV, EPC, CEC, interleukin 6 (IL-6), and other potential biomarkers.

**Results:**

NOV and EPCs were independently associated with the oxygen desaturation index (ODI) after adjusting for potential confounders including body mass index (BMI), age, and sex (NOV *p* = 0.032; EPC *p* = 0.001). EPC was also independently associated with AHI after adjusting for BMI, age, and sex (*p* = 0.017). IL-6 was independently associated with AHI, but not with ODI.

**Conclusion:**

NOV and EPC levels correlate with the degree of OSA independent of BMI, indicating that these biomarkers could potentially further elucidate the relationship between OSA patients and their risk of the subsequent development of cardiovascular disease.

## 1. Introduction

Obstructive sleep apnea (OSA) is a highly prevalent disorder, ranging from 3% to 17% in the general population depending on age and gender [1]. OSA is characterized by repetitive episodes of upper airway closure resulting in a reduction or complete cessation of airflow and intermittent hypoxia; obstructive respiratory events are terminated with an arousal state accompanied by sympathetic surges [2]. The result of poor alveolar ventilation associated with apnea/hypopnea events reduces arterial oxygen saturation and increases arterial pressure of carbon dioxide causing intermittent hypoxia. This leads to oxidative imbalance and increased inflammatory cytokines, lipid peroxidation, and cell-free DNA [3]. The severity of OSA is quantified by overnight sleep studies which measure the apnea-hypopnea index (AHI) and oxygen desaturation index (ODI). Risk factors for OSA include high body mass index, male gender, and age, resulting in a patient population already at risk for cardiometabolic disease. Indeed, OSA has been associated with prevalent and incident hypertension [4, 5], coronary artery disease, and cerebrovascular events [6], likely via inflammatory processes from oxidative stress with increased reactive oxygen species (ROS) formation and proinflammatory cytokines [7]. However, studies associating nephroblastoma overexpressed (NOV), endothelial progenitor cells (EPC), and circulating endothelial cells (CEC) with OSA and as a potential measure of vascular inflammation to determine the risk for cardiovascular disease (CVD) in OSA patients are minimal (NOV) or discrepant (EPC/CEC). A recent meta-analysis showed a linear correlation between AHI severity and olfactory dysfunction, but statistical differences between mild-moderate-severe were not seen [8]. Other recent findings include the apelin ligand of G protein-coupled receptor APJ; the apelin/APJ system appears to be closely related to the development of respiratory diseases, including OSA, that may well be an attractive target for therapeutic intervention [9].

NOV is a multifunctional protein that plays a role in inflammation, cancer, and fibrosis through its involvement in adhesion and mitosis pathways [10] and has been associated with multiple disorders either directly or indirectly linked to cardiovascular disease. Previously, we demonstrated a novel association between OSA and NOV in a clinical sample of obese and nonobese subjects **[**11**]**.

CECs and EPCs are involved with vascular injury and repair. CECs are essentially “sloughed endothelial cells” resulting from systemic inflammation, which are replaced by EPCs that are expected to increase as inflammation-induced CECs sloughing increase [12]. When the EPC can no longer sustain the replacement of sloughed CECs, this denuded area is now ripe for plaque formation [13]. In a prior study, we found that CECs in morbidly obese women at increased risk of cardiovascular disease were elevated and EPCs were altered in obesity, suggestive of early inflammation [12]. Another study has also shown CEC elevation in a population of type 2 diabetics, showing that inflammation induced by diabetes was independent of HgbA1C levels [14]. Although these studies do not involve OSA patients, due to the strong association of OSA with both morbid obesity and diabetes, similar associations with OSA are likely present.

In the current study, we sought to demonstrate that in a sample of well-characterized OSA subjects at increased risk for cardiovascular disease, baseline inflammation in OSA, as demonstrated by changes in known and novel inflammatory biomarkers, may help to risk stratify this population and further elucidate a pathogenic link between OSA and cardiovascular disease. This manuscript is a follow-up study to our previous work [11] with a larger sample of subjects and further blood analysis including EPC, CEC, and cytokines. Specifically, we hypothesized that NOV and other inflammatory adipokines, CECs, and EPCs would independently correlate with increasing OSA severity and provide further evidence to support novel pathways leading to endothelial damage and cardiovascular disease.

## 2. Methods

### 2.1. Study Design and Sample

Study subjects and controls were enrolled at New York-Presbyterian Brooklyn Methodist Hospital (NYPBMH), and laboratory analysis of blood samples was analyzed at New York Medical College (NYMC). Subjects were drawn from individuals presenting to the Center for Sleep Disorders at NYPBMH for evaluation of possible OSA and from faculty and staff of NYMBMH who were not at risk for OSA to serve as controls. Subjects were considered for enrollment only in the absence of a known history (chart review) of coronary artery disease, atherosclerosis, or congestive heart failure. Only adults (age > 18 years old) were recruited. All subjects provided informed consent. A total of 61 subjects were enrolled. IRB approval at the clinical site (NYPBMH) was obtained prior to enrollment. All data were collected prospectively.

### 2.2. Clinical Parameters

All recruited patients had a complete history and physical examination. Patient demographics were collected including age, gender, and race. Patients underwent measurement of systolic and diastolic blood pressure, height and weight for BMI determination, neck circumference, and waist and hip circumference for waist-hip ratio determination by standard methods. Patients were also queried, and medication lists were evaluated to determine the presence of comorbid medical conditions (hypertension, diabetes, hyperlipidemia, chronic obstructive pulmonary disease, and asthma).

### 2.3. Polysomnography

All patients underwent nocturnal polysomnography (PSG) either by (1) conventional full-montage in-laboratory PSG or (2) home sleep testing; studies were performed in accordance with American Academy of Sleep Medicine (AASM) guidelines. Conventional full-montage in-laboratory PSG was performed using Compumedics (Victoria, Australia) software: standard 10-20 electroencephalography (EEG), electrocardiography (ECG), electromyography (EMG) of the chin and anterior tibialis muscle, electrooculography (EOG), snore, and pulse oximetry monitoring were utilized. Oral and nasal airflow were measured by pressure transducer and thermocouple. Respiratory effort was measured with respiratory impedance plethysmography bands at the chest and the abdomen including summation channel. Home sleep testing was performed using ResMed ApneaLink Air (San Diego, California). An apnea was defined as a reduction in peak thermal sensor (or nasal pressure signal in the case of home sleep testing) excursion by ≥90% of baseline, in which there is continued or increased inspiratory effort throughout the entire period of absent airflow lasting at least 10 seconds. Desaturation and/or arousal were not required. A hypopnea was defined as an abnormal respiratory event lasting at least 10 seconds with at least a ≥30% reduction in the nasal pressure signal excursion (or alternate sensor) accompanied by a ≥4% oxyhemoglobin desaturation. The AHI is a measure of OSA severity and derived from the number of apneas and hypopneas per hour of sleep (in-lab determination) or per hour of recording time (home sleep testing). OSA was defined as AHI ≥ 5/hr for analysis purposed. Further analysis with AHI ≥ 15/hr was also explored.

### 2.4. Laboratory Measurement

Venous blood was drawn from antecubital vein into a serum separator tube and a tube containing EDTA. Each SST sample was centrifuged at a force of 1600 g for 10 minutes after blood draw. The tubes were placed in an insulated container with dry ice until analysis.

### 2.5. Plasma NOV Protein Levels

Subjects frozen plasma was suspended in buffer (mmol/l: 10 phosphate buffer, 250 sucrose, 1.0 EDTA, 0.1 PMSF, and 0.1% v/v tergitol, pH 7.5). Immunoblotting for NOV was performed as previously described [15]. NOV levels were based on densitometry fold-increase from a single control sample.

### 2.6. Blood Samples and Cytokine Measurements

After overnight fasting, venous blood was drawn from an antecubital vein to measure serum levels of inflammatory cytokines, Leptin, and EPC testing (blood was drawn in heparinized tubes). Serum samples were frozen at -80°C before analysis. IL-6 was determined using ELISA.

### 2.7. Isolation of Circulating Endothelial Cells

A 10 ml sample of peripheral blood was obtained and used for CEC and EPC experiments. One ml of blood was incubated with 100 *μ*l of anti-CD146 coated 45 *μ*m Dynabeads (1.4 × 108 beads/ml) overnight at 4°C in a Dynal mixer (Dynal, Lake Success, New York) at 50 rpm. Cells bound to anti-CD146 coupled beads were separated from blood in a Dynal magnet, washed (3 washings using phosphate-buffered saline and 0.1% bovine serum albumin and repetitive mixing for 5 minutes in the Dynal mixer at 4°C), and dissolved in 100 *μ*l buffer. Side-by-side assays were performed with Dynabeads coated with human antibodies against mouse IgG but without an antiendothelial antibody to check for nonspecific binding to the Dynabeads. The cells were mixed with acridine and visualized by light and fluorescence microscopy.

### 2.8. Isolation of Mononuclear Cells and EPC Colony Formation

Peripheral mononuclear cells (PMNCs) were fractionated using Ficoll density-gradient centrifugation. Isolated PMNCs were resuspended in CFU-Hill Medium (Stem Cell Technologies, Vancouver, Canada) and plated on 6-well plates, coated with human fibronectin at a concentration of 5 × 10^6^ cells per well. After 48 hours, the nonadherent cells were collected and replated onto fibronectin-coated 24-well plates. EPC colonies were counted using an inverted microscope 7 days after plating. An EPC colony was defined as a cluster of at least 100 flat cells surrounding a cluster of rounded cells, as previously described [12]. Results are expressed as the mean number of colony-forming units (CFUs) per well.

### 2.9. Statistical Analysis

We summarized continuous variables using means and standard deviations and summarized categorical variables as frequencies and percentages. Continuous variables were compared with Student's *t*-test or Mann–Whitney/Wilcoxon paired test as appropriate. Categorical variables were compared with the chi-square test for independence. The relationship between NOV and OSA was compared in several different categories including ODI quartiles, AHI quartiles, and categories of OSA (no OSA, mild OSA, moderate OSA, and severe OSA) as defined by the AASM; these relationships were determined both between groups and overall trends. Due to nonnormal distribution of dependent variables, the cube-root transformation of NOV (and ODI/AHI when dependent variables), which approximated normality, was used in both multivariable models and between group comparisons (Student's *t*-test) to ensure validity of the model. All analyses were performed in Stata 15.1.

## 3. Results

61 subjects were recruited for enrollment. 3 subjects did not complete sleep studies following blood draw. In the overall group, the mean age was 42.6 years. Patients with OSA were significantly older than those without OSA. Women predominated the study population (57%). There was a similar proportion of OSA among men and women (66%). Black race accounted for 54% of the study population, while white race accounted for 31%. Among comorbid conditions, hypertension was the most common at 31%; hypertension was seen more frequently in OSA vs. no OSA subjects (41% vs. 11%, *p* = 0.018). BMI was greater in OSA vs. no OSA subjects (42.9 ± 10.4 vs. 32.1 ± 11.8 kg/m^2^; *p* = 0.007). 67% of subjects demonstrated OSA on sleep testing with an AHI ≥ 5/hr while 41% of subjects had OSA under more stringent criteria of AHI ≥ 15/hr (Table 1).

**Figure 1:**
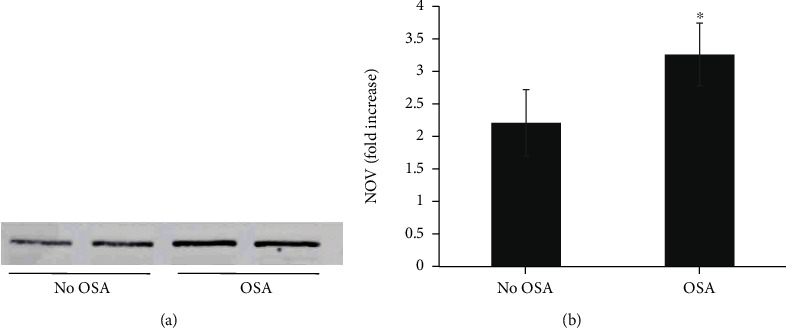
(a) Representative serum immunoblotting analysis for NOV in no OSA vs. OSA subjects. Subjects with OSA displayed an increase in NOV proteins on western blot when compared to those without OSA. (b) Fold change in NOV expression in OSA vs. no OSA subjects. Subjects with OSA displayed greater fold-increase in NOV levels when compared to subjects without OSA (*n* = 50, ^∗^*p* = 0.02). Results are mean ± SE.

**Figure 2:**
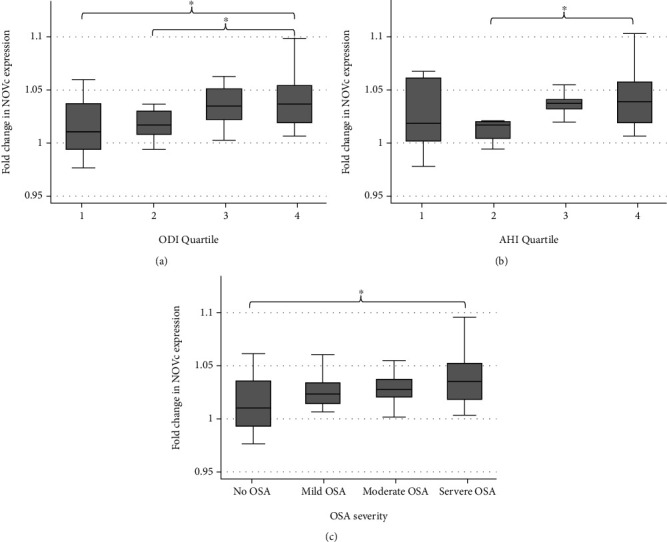
Box plots demonstrating change in cube-root transformed NOV levels (NOVc) as severity of sleep apnea increases. (a) ODI quartiles, overall trend *p* = 0.002; (b) AHI quartiles, overall trend *p* = 0.039; (c) OSA severity, overall trend *p* = 0.013. ^∗^*p* < 0.05. These plots were made using cube root transformation of NOV and Student's *t*-test performed in Stata 15.1. (a) Box plots representing NOV levels in the different ODI quartiles. Subjects in the upper ODI quartiles have increasing levels of NOV. There is statistically significant higher expression of NOV in ODI quartile 4 when compared to ODI quartiles 1 and 2. The overall trend for NOV expression increases as ODI quartile increases. (b) Box plots representing NOV levels in the different AHI quartiles. Subjects in the upper AHI quartiles have increasing levels of NOV. There is statistically significant higher expression of NOV in AHI quartile 4 when compared to AHI quartile 2. The overall trend for NOV expression increases as AHI quartile increases. (c) Box plots representing NOV levels in the different severities of OSA. Subjects in the high severity groups have increasing levels of NOV. There is statistically significant higher expression of NOV in the severe OSA group when compared to the no OSA group. The overall trend for NOV expression increases as AHI quartile increases.

NOV levels were greater among those with OSA (3.3 ± 2.9 vs. 2.2 ± 2-fold increase in OSA vs. no OSA (*p* = 0.02) (Figure 1)). NOV levels increase as quartiles of ODI (*p* = 0.002) and AHI (*p* = 0.039) increase. Within group differences were observed (Figure 2): NOV was different among ODI quartiles 1 and 3 (*p* = 0.028), quartiles 1 and 4 (*p* = 0.009), and quartiles 2 and 4 (*p* = 0.033), while NOV was different among AHI quartiles 2 and 4 (*p* = 0.033). NOV levels increase as OSA severity categories (clinical severity of no OSA, mild, moderate, and severe OSA) increase (*p* = 0.013); within group differences in NOV were only seen comparing mild and severe OSA (*p* = 0.011).

**Figure 3:**
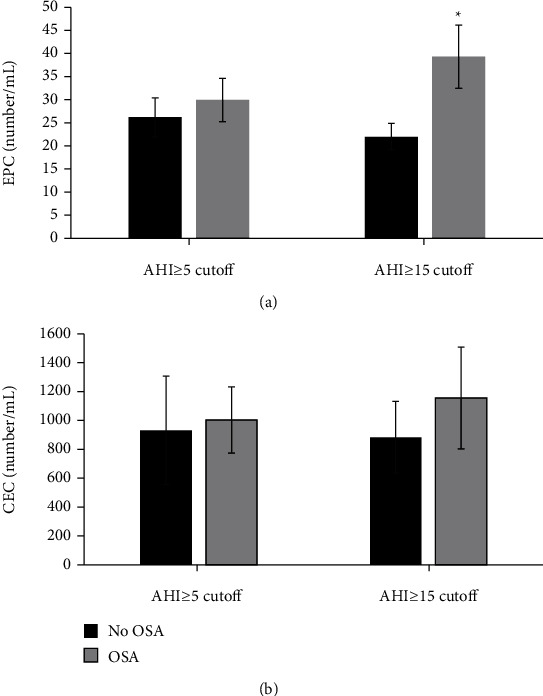
(a) Difference in EPC levels in subjects with OSA vs. no OSA; OSA defined as AHI ≥ 5/hr or AHI ≥ 15/hr. Those with OSA had higher EPC levels when more stringent criteria for OSA diagnosis (i.e., AHI ≥ 15) were used supportive that more severe disease has an association with higher EPC levels (*n* = 53, ^∗^*p* = 0.021). (b) Difference in CEC levels in subjects with OSA vs. no OSA; OSA defined as AHI ≥ 5/hr or AHI ≥ 15/hr. Those with OSA displayed higher levels of CEC, but the association did not reach statistical significance (*n* = 44).

**Figure 4:**
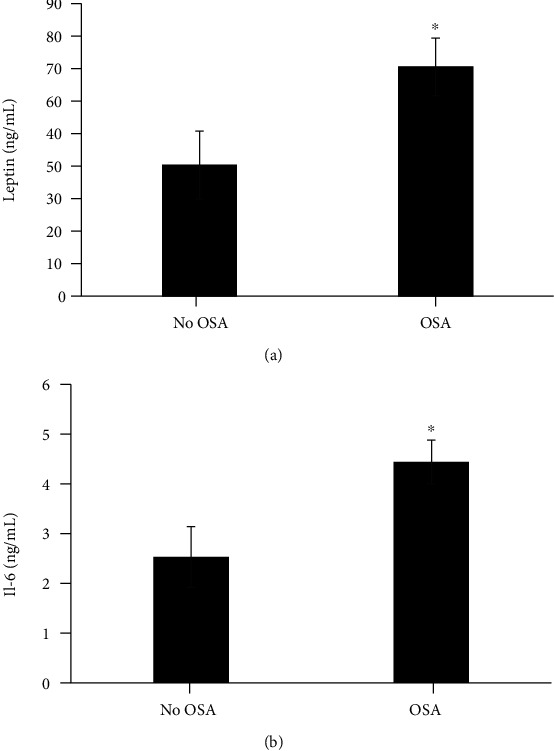
(a) Difference in Leptin levels by OSA vs. no OSA. Those with OSA had higher levels of leptin (*n* = 59, ^∗^*p* ≤ 0.05) prior to adjusting for age, gender, and BMI. This association was lost when BMI was adjusted for indicating BMI is a strong confounding variable. Results are mean ± SE. (b) Difference in IL-6 levels by OSA vs. no OSA. Those with OSA had higher IL-6 levels (*n* = 59, ^∗^*p* ≤ 0.05). This association was seen in both ODI and AHI as parameters for diagnosing OSA prior to adjusting for BMI. When BMI was added IL-6 maintained association with AHI only (*p* = 0.049) supportive that BMI is a confounding variable. Results are mean ± SE.

EPC and CEC were not significantly different when comparing OSA vs. no OSA (Figure 3), while leptin and IL-6 were (leptin: 70.5 ± 54 vs. 40.3 ± 45.5 ng/mL, *p* = 0.015; IL-6 4.4 ± 2.7 vs. 2.5 ± 2.7 ng/mL, *p* = 0.006; Figure 4). EPC levels increased as quartiles of ODI increased (*p* = 0.017) but not with AHI quartiles or by OSA severity categories. In exploratory analysis, EPC levels were higher in OSA when OSA was classified as AHI ≥ 15 hr (39.3 ± 29.8 vs. 22 ± 16.1 number/mL, *p* = 0.021) (Figure 3(a)). These results support that more severe disease has an association with higher EPC levels.

In multivariable analysis (Table 2), after adjusting for age, gender, and BMI, NOV was independently associated with ODI (*p* = 0.032). NOV was not independently associated with AHI. EPCs were independently associated with both ODI (*p* = 0.001) and AHI (*p* = 0.017). Leptin was independently associated with both ODI and AHI in model 2; however, when BMI was added, leptin was no longer associated with either parameter indicative that BMI is a strong confounding variable. IL-6 was independently associated with ODI (*p* < 0.0001) and AHI (*p* < 0.0001) in model 2; however, when BMI was added, it was no longer associated with ODI but maintained significance with AHI (*p* = 0.049), which supports that BMI confounded IL-6 as well.

## 4. Discussion

We have shown for the first time that measures of obstructive sleep apnea (OSA) are independently associated with the novel adipokine matricellular protein nephroblastoma overexpressed (NOV) and endothelial progenitor cells (EPC) after adjusting for baseline demographics and BMI. Specifically, NOV levels were higher in those with OSA compared to those without OSA in univariate analysis. Further, multivariable methods showed that NOV is associated with the oxygen desaturation index (ODI) after adjusting for age, gender, and BMI; this finding was not seen in association with the apnea hypopnea index, suggesting that intermittent hypoxia, as specifically measured by the ODI, is central to this relationship, and that the more general measure of sleep-disordered breathing (AHI), which may also include nonhypoxic arousal events, is not. EPCs, in contrast, are independently associated with both the ODI and AHI, suggesting that overall mechanisms of OSA including intermittent hypoxia and other pathophysiologic variables are important in this relationship. Leptin and IL-6 levels related to OSA measures appear to be modified by BMI.

Inflammation in OSA is driven by intermittent hypoxia and fragmented sleep leading to oxidative stress, manifested by increased formation of ROS and elevated adipocytokines [16, 17]. Oxidative imbalance is the result of this intermittent hypoxia with increased inflammatory cytokine production of IL-6, TNF, and lipid peroxidation; literature review has not identified which biomarkers better correlate with the severity of disease [3]. CPAP has been shown to decrease this oxidative stress [3] and normalize ROS, nitric oxide, and 8-isoprostane levels [18]. In contrast, obesity's baseline chronic inflammatory state is due to insulin and leptin resistance resulting in increased inflammatory cytokines released from white adipose tissue (WAT), which has diminished thermogenic capability from mitochondrial dysfunction compared to brown or beige adipose tissue (BBAT) [19–21]. Due to OSA's strong correlation with obesity, the inflammation and associated comorbid conditions of obesity can be seen in OSA patients. Our findings suggest that obesity plays a role in chronic inflammation (as seen in leptin and IL-6 levels modified by BMI) while the additional inflammatory pressure resulting in elevated NOV and EPC levels is likely driven by intermittent hypoxia and inflammation specific to OSA pathophysiology. The development of atherosclerosis is thought to be highly influenced by this intermittent hypoxia; this involves a complex interaction of multiple factors that include oxidative stress and inflammation, autonomic nervous system dysfunction, and platelet activation [22].

NOV, a member of the CCN multifunctional proteins, plays a role in inflammation, cancer, and fibrosis through its involvement in adhesion and mitosis pathways [10]. It has been associated with multiple disorders either directly or indirectly linked to cardiovascular disease. NOV is an established regulator of and regulated by various cyto/chemokines, including the anti-inflammatory enzyme heme oxygenase-1 (HO-1) [23], [10]. A recent study in humans showed that NOV is strongly correlated with BMI and fat mass, decreases with weight loss, and is associated with elevated hemoglobin A1c levels [24]. Disease states associated with increased inflammation, including endothelial cell dysfunction, obesity, insulin resistance, metabolic syndrome, and interstitial renal fibrosis, have all been associated with increased NOV levels [23–25], [26]. Furthermore, epoxyeicosatrienoic acid (EET), a molecule that inhibits the inflammatory process, improves insulin sensitivity, and decreases NOV was shown to attenuate obesity-induced cardiomyopathy by down regulating NOV, increasing heme oxygenase-1 (HO-1) and Wnt signaling in both cardiac and pericardial fat [15, 27]. This resulted in decreased inflammatory cytokines IL-6 and TNF, as well as an increase in anti-inflammatory molecules and mitochondrial integrity [15, 27]. HO-1 upregulation and EET upregulation both result in marked reductions of IL-6, TNF, and NOV [28]. The knockout mouse model of peroxisome proliferator-activated receptor gamma coactivator-1*α* (PGC-1 *α*) reversed these findings and blocking the nuclear coactivator of HO-1 suggested that HO-1 upregulation was the mechanism involved. These findings propose that NOV may play a role in the OSA-related risk of developing cardiac disease and may be a target for potential therapy. This was shown again by administration of an EET agonist that increased PGC-1 *α*, which induced the HO-1 increase, improved mitochondrial function, and induced a change in the pericardial and epicardial adipocyte phenotype from white to beige; the improved insulin receptor phosphorylation improved insulin sensitivity and resulted in reversal of heart failure [27]. Thymoquinone (TQ) is another molecule that comes from the Nigella sativa plant that has major anti-inflammatory properties; in combination with omega fish oils, they improved insulin sensitivity in obesity and promoted the browning of white fat, with upregulation of mitochondrial enzymes, HO-1 levels, and reduction of the inflammatory adipokine NOV, twist-related protein (TWIST2), and the adipocyte hypoxia inducible factor HIF-1*α* [29]. NOV induces cytokine formation by increasing adipogenesis, with decreased numbers of mitochondria and diminished mitochondrial function, which is all reversed by EET upregulation [27]. White adipose tissue has higher NOV levels than beige and brown adipose tissue [15]. In addition, the inflammatory state is destructive to mitochondria and contributes to multiorgan dysfunction [30–32]. The ability to generate thermogenesis via mitochondrial electron transport chain (ETC) uncoupling is highest in brown fat, followed by beige, and finally least in white fat [19, 20]. Therefore, lean individuals with a higher brown/beige adipose tissue (BBAT) to WAT ratio have better anti-inflammatory mechanisms to fight severe inflammation due to stable thermogenesis secondary to higher mitochondria concentration, which utilizes HO-1 to promote uncoupling [29, 33].

Other potential predictive markers such as CECs and EPCs are of interest in proinflammatory disease states as well. CECs are accepted and reliable indicators of vascular damage [34]. CECs are a measure of “sloughed cells” from the vascular endothelium that are routinely replaced by bone marrow-derived EPCs as part of the injury-repair mechanism, but EPCs are rarely identified in the peripheral blood of healthy individuals, and when they are, they will be few in number [35]. When the production of EPCs are no longer able to replace the increasing amounts of sloughed CECs, the area of denuded endothelium is now primed for the development of vascular plaques [36]. EPCs have been implicated in plaque “vulnerability to rupture” [12, 37]. As a result, their presence in peripheral blood is a measure of endothelial injury/repair [34]. Endothelial dysfunction is the initial stage of atherosclerotic disease and more need to be done to identify patients at increased risk and intervene [38].

Quantification of CEC and EPC has correlated with cardiovascular disease. Studies show increased levels of CECs in the acute coronary syndrome spectrum, stable angina, ischemic cerebrovascular accident (CVA), and critical limb ischemia [39]. CEC quantification 48 hours after acute coronary syndrome was shown to accurately risk stratify patients for major adverse coronary events and death at both one month and one year [39]. Furthermore, the pathogenesis of atherosclerosis and plaque rupture leading to ischemic events is a proinflammatory process resulting in higher oxidative stress in the endothelium [40]. On the other hand, decreased EPCs have been associated with increased levels of cardiovascular disease [36]. One theory posits that there is a finite supply of EPCs, and once they have been exhausted due to repetitive vascular injury, the evolution of cardiovascular disease is established [41].

Individuals with higher levels of oxidative stress, such as those with OSA and obesity, have higher risk for unstable plaque formation over the damaged endothelium that consists of cholesterol deposits and foam cells [40]. Presumably, those with established cardiovascular disease would demonstrate a reduction in EPCs, and this should be in a dose response manner to the severity of OSA. However, this was not what we found in our study, and the literature in OSA and EPCs is quite variable. Multiple investigators have found that EPCs are reduced in those with OSA [42]; [43]; [44]. Alternatively, other investigators found that EPCs were increased [45] or unchanged [46] [47] in OSA vs. control. Potential reasons for these discrepant results are numerous, including differences in EPC measurement, differences in patient population (i.e., race and age) [48], and differences in the presence and evolution of cardiovascular disease in each individual patient. Those with changes in EPC levels are at risk for a vulnerable thin fibrous cap rupture leading to acute thrombosis placing individuals with OSA at higher risk for cardiovascular disease [12, 37, 40]. Intermittent hypoxia seen in OSA causes increased ROS formation as a result of decreased oxygenation and may subsequently result in higher EPC levels as the endothelium undergoes repair [7, 40]. In our study, EPC levels were found to be independently associated with both ODI (*p* = 0.001) and AHI (*p* = 0.017) (Table 2). There was no statistically significant correlation between OSA and CEC in our data; of note, only 44 subjects had CECs measured in this study which may have resulted in a type 2 error.

The proinflammatory state of OSA was confirmed in our subjects by increased levels of IL-6. There was an overall positive correlation between AHI and IL-6 (Table 2, model 1 *p* = 0.003), but the relationship was weakened when adjusted for BMI (Table 2 model 3 *p* = 0.049), suggesting the inflammatory state of obesity acts as an effect modifier. Similar findings are well established in OSA patients, in which several inflammatory markers are known to be elevated in OSA, including leptin, CRP, TNF-a, and IL-6 [49].

There are several limitations in our study. Although we feel that the findings are generalizable, the fact that we had a large percentage of black subjects makes this less so. Known racial differences in cardiovascular disease [50] may indicate that the pathogenic mechanisms underlying the vascular inflammatory cascade may also be different. Because our study subjects in general did not have established coronary or other cardiovascular diseases, we were unable to differentiate those in whom EPCs may have been decreased due to exhaustion of the bone marrow response from those with an exuberant EPC response. Because we did not collect actigraphy data, we were unable to assess the role that shortened sleep duration and therefore sleep deprivation may have played in our results. Similarly, the lack of arterial blood gas analysis to determine the presence of hypoventilation prevented us from identifying subjects with obesity-hypoventilation syndrome, which may affect the degree of deoxygenation and thus oxidative stress. Finally, the cross-sectional nature of our study prevents any temporality and therefore any causal inference.

## 5. Conclusion

The subjects of our study demonstrated that OSA is independently correlated with NOV after adjusting for BMI, age, and sex when compared to control. NOV appeared to be driven by intermittent hypoxia rather than general obstructive episodes as the correlation was with ODI and not AHI. EPCs were independently associated with both ODI and AHI, while CECs did not demonstrate an association with OSA. IL-6 was elevated in OSA subjects based on AHI, but BMI appeared to be a strong modifier of this relationship. Leptin was not associated with OSA after fully adjusting for BMI. In summary, the inflammatory adipokine NOV and EPC represent potential biomarkers that may help identify OSA patients at a current or future increased risk of cardiovascular disease from oxidative stress and may be a potential target to prevent the vascular downstream consequences of this systemic inflammatory cascade.

## Figures and Tables

**Table 1:** Baseline characteristics^∗^.

	Total (*n* = 61)	No OSA (*n* = 19)	OSA (*n* = 39)	*p* value
Age (mean)	42.6 ± 13.8	34.6 ± 8.7	45.4 ± 12.8	*p* = 0.0016
Gender (%)				NS
Men	26 (43)	8 (42)	16 (41)	
Women	35 (57)	11 (58)	23 (59)	
Race (%)				NS
White	19 (31)	7 (37)	10 (36)	
Black	33 (54)	7 (37)	25 (64)	
Hispanic	3 (5)	0	3 (8)	
Asian	6 (10)	5 (26)	1 (3)	
Comorbidities (%)				
Hypertension	19 (31)	2 (11)	16 (41)	*p* = 0.018
Diabetes	13 (21)	2 (11)	10 (26)	NS
Hyperlipidemia	10 (16)	1 (5)	8 (21)	NS
BMI (kg/m^2^)	39 ± 11.9	32.1 ± 11.8	42.9 ± 10.4	*p* = 0.0007
AHI (events/hr)	21.4 ± 30	2.4 ± 1.3	30.6 ± 32.9	*p* = 0.0004
ODI (desat/hr)	17.3 ± 24.7	2.3 ± 2.0	24.6 ± 27.3	*p* = 0.0008
OSA (≥5/hr) (%)	39 (67)			
OSA (≥15/hr) (%)	24 (41)			

Abbreviations: OSA: obstructive sleep apnea; BMI: body mass index; AHI: apnea hypopnea index; ODI: oxygen desaturation index; NS: not significant (*p* > 0.05). ^∗^Note: 3 subjects did not complete sleep study, accounting for the discrepancy between total and subgroup frequency.

**Table 2:** Regression model with dependent variables (ODI and AHI) vs. independent variables.

	Model 1	Model 2	Model 3
ODIc			
NOVc	17.1 (4.8, 29.3) ¶	20.0 (7.5, 32.6) ¶	14.4 (1.3, 27.5)^∗^
EPCs	0.16 (0.03, 0.29)^∗^	0.17 (0.04, 0.31)^∗^	0.20 (0.09, 0.31) ¶
Leptins	0.07 (-0.01, 0.16)	0.13 (0.05, 0.22) ¶	0.05 (-0.06, 0.16)
IL-6 s	0.57 (0.22, 0.93) ¶	0.71 (0.35, 1.1)^§^	0.46 (-0.10, 1.0)
AHIc			
NOVc	11.0 (-1.6, 23.6)	12.9 (-0.1, 25.9)	7.4 (-6.2, 21.1)
EPCs	0.11 (-0.01, 0.24)	0.12 (-0.01, 0.26)	0.14 (0.03, 0.26)^∗^
Leptins	0.05 (-0.04, 0.13)	0.10 (0.01, 0.19)^∗^	0.02 (-0.10, 0.13)
IL-6 s	0.54 (0.19, 0.90)^¶^	0.68 (0.33, 1.03)^§^	0.56 (0.002, 1.11)^∗^

Model 1 is before adjustment; model 2: adjustment for age and sex; model 3: adjustment for BMI, age, and sex. ^∗^*p* < 0.05; ^¶^*p* < 0.01; ^§^*p* < 0.0001. Abbreviations: ODIc: cube-root transformed oxygen desaturation index; AHIc: cube-root transformed apnea hypopnea index; NOVc: cube-root transformed NOV levels; EPCs: square-root transformed endothelial progenitor cells; Leptins: square-root transformed Leptin; IL-6 s: square-root transformed interleukin-6.

## Data Availability

Access to data is restricted due to legal and ethical concerns.

## Additional Points

*Study Importance Questions*. What is already known about this subject? (i) OSA is associated with oxidative stress of chronic inflammation, elevation of adipocytokines, and endothelial dysfunction. (ii) OSA is associated with cardiometabolic disease. What are the new findings in your manuscript? (i) NOV levels correlate with the degree of OSA independent of BMI. (ii) EPC levels correlate with the degree of OSA independent of BMI. How might your results change the direction of research or the focus of clinical practice? NOV and EPC represent potential biomarkers which may help to identify OSA patients at a current or future increased risk of cardiovascular disease caused by oxidative stress and chronic inflammation and may be a potential target to prevent the vascular downstream consequences of this systemic inflammatory cascade.
